# Capturing metabolic syndrome in low-resource settings: a case study in urban Haiti

**DOI:** 10.3389/fendo.2025.1651058

**Published:** 2025-09-11

**Authors:** Nour Mourra, Vanessa Rouzier, Rodney Sufra, Reichling St Sauveur, Jodany Bernadin, Joseph Inddy, Alexandra Apollon, Rehana Rasul, Anju Ogyu, Lily D. Yan, Jean W. Pape, Margaret McNairy

**Affiliations:** ^1^ Doctor of Medicine Program, Department of Medicine, Emory University School of Medicine, Atlanta, GA, United States; ^2^ Center for Global Health, Department of Medicine, Weill Cornell Medicine, New York, NY, United States; ^3^ Haitian Group for the Study of Kaposi’s Sarcoma and Opportunistic Infections (GHESKIO), Port-au-Prince, Haiti; ^4^ Department of Epidemiology and Biostatistics, Graduate School of Public Health and Health Policy, City University of New York, New York, NY, United States; ^5^ Division of General Internal Medicine, Department of Medicine, Weill Cornell Medicine, New York, NY, United States

**Keywords:** metabolic syndrome, LMIC, cardiovascular disease, insulin resistance, elevated waist circumference, hypertension

## Abstract

**Introduction:**

Local epidemiologic data on risk factors for heart disease are needed in low-income settings to guide targeted interventions for prevention and treatment. Metabolic syndrome (MetS) is a cluster of conditions (elevated blood pressure, blood sugar, waist circumference and cholesterol) that increases risk for cardiovascular disease (CVD), however the gold-standard MetS definition requires laboratory testing which are be limited in low-income countries like Haiti. The objective of this study was to estimate the prevalence of MetS in urban Haiti and compare it to alternative nonlaboratory MetS definitions.

**Methods:**

This study is a cross-sectional analysis of enrollment data from the population-based Haiti CVD Cohort Study which includes 3,005 participants ≥18 years, in Port-au-Prince. Demographic, health behavior, and clinical data including laboratory tests were collected. Gold standard, harmonized MetS (MetS-H) was defined as having three or more of the following: elevated blood pressure (eBP), elevated waist circumference (eWC), elevated fasting glucose, reduced HDL-C or elevated triglycerides. Three nonlaboratory alternatives were defined as: MetS-1 (eBP, and eWC), MetS-2 (three or more of: eBP, eWC, family or personal history of CVD), and MetS-3 (four or more of: eBP, eWC, family or personal history of CVD, high alcohol intake, current/former smoker, high fat intake). Sensitivity and specificity were calculated for each nonlaboratory MetS definition, compared to MetS-H. Associations between risk factors and MetS-H were assessed using multivariable log-binomial regressions.

**Results:**

Among 2721 participants with a mean age of 42 years (SD 16), the prevalence of MetS-H was 21.2% (29.1% women, 10.4% men). Elevated blood pressure (82.9%), reduced HDL-C (81.7%) and elevated waist circumference (90.7%) were the most common components of MetS. The prevalence of nonlaboratory definitions were: MetS-1 22.5%, MetS-2 22.6%, and MetS-3 22.2%. Compared with MetS-H, MetS-1 had the highest sensitivity (74.4%, 95% CI: 70.6%, 77.9%) and the highest specificity (91.6%, 95% CI: 90.7%, 92.7%). Female sex and age >30 years were associated with MetS-H.

**Discussion:**

The prevalence of MetS is high in urban Haiti and associated with older age and females. Simplified screening with nonlaboratory MetS definitions may be a pragmatic alternative to screening in low-income countries.

## 1 Introduction

Cardiovascular disease (CVD) is the leading cause of death globally, with the greatest burden in low- and middle-income countries (LMICs) (1). Haiti is an LMIC with a growing CVD epidemic, with modeling studies estimating that CVD accounted for 26% of adult deaths in 2019 (2). Our prior work from the Haiti CVD Cohort Study demonstrated a high prevalence of individual CVD risk factors, such as hypertension (29%) and obesity (17%), for metabolic disorders (3–5).

Metabolic syndrome (MetS) is a cluster of conditions that occur simultaneously and are associated with future CVD and mortality (6, 7). The 2009 harmonized definition of MetS requires three out of five criteria: elevated blood pressure, elevated blood sugar, elevated waist circumference, elevated triglycerides and low HDL-C (8). MetS is associated with accelerated atherosclerosis, myocardial infarction, stroke recurrence, and type 2 diabetes (8–10). It is estimated that a quarter of the population worldwide has MetS (7). Screening for and diagnosing MetS is crucial for identifying targets of intervention for CVD prevention. However, the prevalence of MetS in Haiti and many LMICs is unknown due to lack of systematic, population-based sampling. Furthermore, the gold-standard harmonized definition of MetS requires laboratory testing, which is often limited in low-income settings.

To address this gap, we 1) estimated the prevalence of MetS using population-based cross-sectional enrollment data from the Haiti CVD Cohort Study, and 2) developed nonlaboratory definitions and compared them to the gold-standard harmonized definition as pragmatic alternatives for use in low-resource settings.

## 2 Materials and methods

### 2.1 Study setting and design

**Figure 1:**
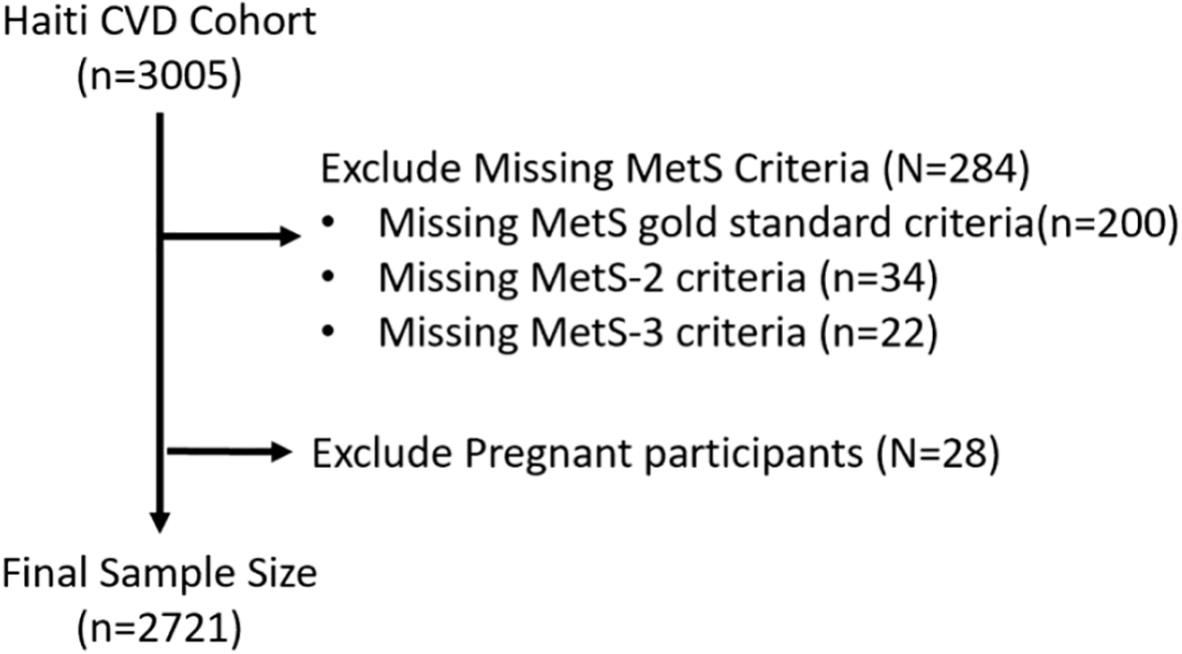
Study flow chart.

We analyzed cross-sectional enrollment data from the population-based Haiti CVD Cohort, which follows 3005 adults in Port-au-Prince longitudinally to understand the prevalence and incidence of CVD (11). Participants were recruited using multistage random sampling from census blocks in Port-au-Prince, with inclusion criteria being over 18 years of age and able to consent (11). A sample size of ~3000 was selected based on power calculations to detect a minimum odds ratio of 1.35 for risk factors and outcomes of hypertension and CVD, assuming 80% power and a two-tail alpha of 0.01 to adjust for multiple comparisons. A protocol paper with more details was published previously (11). The study was conducted at the Groupe Haitien d’Etude de Sarcome de Kaposi et de Infections Opportunistes (GHESKIO) clinic, located in Port-au-Prince. Enrollment in the cohort began March 19, 2019, and concluded August 23, 2021. Participants who had missing data regarding MetS criteria and participants who were pregnant at enrollment were excluded from this analysis (n=284) (**Figure 1** Study flow chart).

### 2.2 Measurements

Self-reported sociodemographic data (age, sex, and education) and health behaviors (smoking status; alcohol consumption; consumption of fruits, vegetables and fats; and physical activity) were collected via the WHO STEPS questionnaires (standard 18–69 years) (12). For income, participants were asked how much they earned in a typical day in the local currency, and this was converted into USD using the conversion rate at time of data collection and converted into a categorical variable of <$1USD/day, $1-$10 USD/day, and >$10USD/day. Smoking was categorized as current/former or never, alcohol as high (≥5 times a week) or low (< 5 times a week), and diet as high in fruits and vegetables (≥ 5 servings a day) or low (<5 servings a day). Physical inactivity was categorized as not doing any manual work or recreational sports. Participants’ oil and fat consumption was categorized as high (usually or always added to food by themselves or food preparers) or low (never or seldom added).

Past medical history of CVD, medication use, and family history of CVD were recorded during the clinic visit. Unattended clinic blood pressure was measured via the OMRON HEM 907 automated oscillometric blood pressure machine following the guidelines of the WHO and the American Heart Association (AHA), which indicates that the participant should be seated in a quiet room for 3–5 minutes with legs uncrossed and both feet on the ground and the arm supported at the heart level (12, 13). The average of the second and third blood pressure measurements was used as the average blood pressure in the analysis. Waist circumference was measured in centimeters via a soft medical measuring tape following the WHO STEPS procedures, positioned at the midpoint of the last palpable rib and the top of the hip bone (12). Laboratory measures included enzymatic plasma glucose (mg/dL) (fasting and nonfasting for those who were unable to fast prior to laboratory tests), high-density lipoprotein cholesterol (HDL-C) (mg/dL), and triglycerides (mg/dL). During survey creation, data entry for clinical values were restricted to positive numbers and biologically plausible entries (SBP 40 to 300 mmHg, DBP 30 to 200 mmHg, waist circumference 30 to 300 cm, plasma glucose above 0 mg/dL, HDL-c 0 to 200 mg/dL, triglycerides 0 to 1000 mg/dL).

### 2.3 Metabolic syndrome definitions

Despite the general agreement of the main components of MetS in the medical community, MetS has been defined differently over the years. For our analysis, we used the 2009 harmonized definition of metabolic syndrome (MetS-H) as the gold standard, which was released by the International Diabetes Federation (IDF) Task Force on Epidemiology and Prevention, the National Heart, Lung, and Blood Institute, the AHA, the World Heart Federation, the International Atherosclerosis Society and the International Association for the Study of Obesity (8). A recent study revealed this to be the best screening tool for MetS compared with prior criteria by the IDF, the Adult Treatment Panel III of the National Cholesterol Education Program and the World Health Organization (WHO) (14).

MetS-H requires three or more of the following criteria: 1) elevated systolic blood pressure (SBP) (≥ 130 mmHg) or elevated diastolic blood pressure (DBP) (≥ 85 mmHg) or being on antihypertensive treatment; 2) elevated waist circumference categorized using population and country-specific definitions; 3) elevated plasma glucose (≥100 mg/dL fasting) or treatment for high blood glucose/diabetes; 4) reduced HDL-C (<40 mg/dL in males or <50 mg/dL in females) or on treatment for cholesterol; and 5) elevated triglycerides ≥150 mg/dL. We used waist circumference thresholds on the basis of the IDF recommendations for sub-Saharan Africans (≥94 cm for men, ≥80 cm for women) (8). For participants who were unable to fast before their labs, we adjusted the threshold for elevated plasma glucose to be ≥200 mg/dL (15).

We also developed three simplified, nonlaboratory-based definitions of MetS in a sequential fashion using measures more readily available in low-resource settings (**Table 1**). From the MetS-H definition, we removed all laboratory values to create the first definition, MetS-1. MetS-1 requires both 1) elevated waist circumference and 2) elevated blood pressure or taking antihypertensive medication, with the same thresholds as the MetS-H definition. MetS-2 was defined as having three or more of the following criteria: 1) MetS**-**1 criteria; 2) past medical history of hypertension, diabetes, hyperlipidemia, myocardial infarction (MI), heart failure (HF), or stroke; and 3) family history of hypertension, MI, HF, stroke, or cardiac death. Past medical history of CVD and family history of CVD were incorporated into the MetS-2 definition given they are known risk factors for MetS, and can be assessed through questionnaires (16, 17). The third definition, MetS-3, was defined as having four or more of the following criteria: 1) MetS-2 criteria, 2) reporting a high consumption of fats and oils, and 3) current/former tobacco user or high alcohol use. These measures were chosen because they are known risk factors for MetS, can be easily assessed via self-report on questionnaires, and are readily available through the WHO STEPs questionnaire which is routinely administered in many low-income countries (16, 18). High consumption of fats and oils has been shown in prior literature to be associated with hyperlipidemia (19) while tobacco use has been shown to be associated with hypertension and cardiovascular disease (20). Self-reported health behaviors are recorded using the validated WHO STEPs questionnaire and have known CVD associations in the literature (18). High consumption of fats and oils was defined as usually or always adding butter or oil to food before consumption or as the food preparer.

**Table 1:** Metabolic syndrome definitions.

Characteristics	Clinical Criteria	Metabolic Syndrome Definition			
		MetS-H	MetS-1	MetS-2	MetS-3
Vital signs	Elevated blood pressure: SBP >130 mmHg or DBP ≥85 mmHg OR antihypertensive drug treatment in a patient with a history of hypertension	X	X	X	X
	Elevated waist circumference: Men: >94 cm, Female: >80 cm	X	X	X	X
Self-Reported Medical History	Past medical history of hypertension, high cholesterol, diabetes, myocardial infarction and/or angina, arrhythmia, heart failure, hospitalized for CVD event.	–	–	X	X
	Family history of cardiovascular disease of stroke, hypertension, myocardial infarction and/or angina, heart failure, cardiac death	–	–	X	X
Self-Reported Health Behavior	High consumption of dietary fats/oils (Defined as usually or always adding butter or oil to food before consumption or as the food preparer)	–	–	–	X
	Current/former smoking activity or high consumption of alcohol (≥5 times a week)	–	–	–	X
Laboratory Test	Elevated fasting glucose: ≥100 mg/dL (if fasting), ≥200 mg/dL (if nonfasting) OR on drug treatment of elevated glucose	X	–	–	–
	Reduced HDL-C: Men: <40 mg/dL, Female: <50 mg/dL OR drug treatment for reduced HDL-C	X	–	–	–
	Elevated Triglycerides: >150 mg/dL OR drug treatment for elevated triglycerides	X	–	–	–
	Total score to meet definition	≥3	2	≥3	≥4

High Alcohol Use: defined as ≥5 times a week.

### 2.4 Data analysis

Descriptive statistics were calculated using mean and standard deviation for continuous variables and counts and percentages for categorical variables. The crude prevalence of each definition of MetS was also tabulated overall and stratified by sociodemographic, health behavior, and metabolic risk factors. The age-adjusted prevalence and corresponding 95% confidence intervals (CIs) for each MetS definition were also calculated via direct standardization to the WHO 2000–2025 World Standard Population overall (21).

The sensitivity, specificity, positive predicted value, negative predicted value and corresponding 95% CI based on exact binomial confidence limits were calculated for each nonlaboratory MetS definition (MetS-1, 2 and 3) overall and by sex, with MetS-H as the reference. Cohen’s kappa test was used to determine whether the agreement between the two methods (comparing the gold standard to the nonlaboratory definition) was greater than would be expected by chance (22). A 2x2 table was also generated to compare the different MetS definitions. Analyses were conducted via R, version 4.3.1.

As the primary analysis, crude and multivariable log-binomial regressions were fitted to evaluate the associations of age group, sex, education, poverty level, and smoking status with gold standard MetS-H definition. The log-binomial regression method is the preferred method to calculate the prevalence ratio for cross-sectional analyses as it yields less biased estimates compared to logistic regression (23, 24). As sensitivity analyses, multivariable Poisson regressions to generate prevalence ratios, and multivariable logistic regressions to generate odds ratios, are also presented.

### 2.5 Ethics

Written informed consent was obtained from all the subjects. This study was approved by the Weill Cornell Medicine Institutional Review Board (record number 1803019037) and the Groupe Haitien d’Etude du Sarcome de Kaposi et des Infections Opportunistes (GHESKIO) Comité des Droits Humains. All research was performed in accordance with relevant regulations.

## 3 Results

A total of 2,721 participants were included in the analysis. The mean age was 42 years (SD 16), and 58.0% were women. The majority, 70.1%, of the participants reported an income of less than $1 USD/day, and 63.9% had a secondary education or higher. Most participants (99.4%) reported consuming fewer than 5 servings of fruits and vegetables per day, and nearly half of the participants reported low physical activity (49.4%) (**Table 2**). Among the 2,721 participants, the most prevalent MetS criteria were elevated waist circumference (44.9%), elevated blood pressure (39.0%) and reduced HDL-C (40.6%).

**Table 2:** Participant characteristics in the Haiti cardiovascular disease cohort.

Characteristics	Overall (n = 2,721, 100.0%)	Male (N = 1,143, 42.0%)	Female (N = 1,578, 58.0%)
Age in years, Mean (SD)	42.21 (15.92)	41.22 (16.45)	42.93 (15.48)
Age group			
<30 years	782 (28.7%)	387 (33.9%)	395 (25.0%)
30–39 years	515 (18.9%)	197 (17.2%)	318 (20.2%)
40–49 years	477 (17.5%)	179 (15.7%)	298 (18.9%)
50–59 years	460 (16.9%)	170 (14.9%)	290 (18.4%)
≥60 years	487 (17.9%)	210 (18.4%)	277 (17.6%)
Education			
Less than secondary	1,740 (63.9%)	837 (73.2%)	903 (57.2%)
Secondary or higher	981 (36.1%)	306 (26.8%)	675 (42.8%)
Income			
≤$1 USD/day	338 (12.4%)	140 (12.2%)	198 (12.5%)
$1–10 USD/day	1,907 (70.1%)	819 (71.7%)	1,088 (68.9%)
> $10 USD/day	476 (17.5%)	184 (16.1%)	292 (18.5%)
Fruit/Vegetable Servings			
<5 average servings/day	2,704 (99.4%)	1,141 (99.9%)	1,563 (99.1%)
>5 average servings/day	15 (0.6%)	1 (0.1%)	14 (0.9%)
(Missing)	2	1	1
Smoking			
Current/Former	206 (7.6%)	138 (12.1%)	68 (4.3%)
Never	2,515 (92.4%)	1,005 (87.9%)	1,510 (95.7%)
High Alcohol Use	101 (3.7%)	78 (6.8%)	23 (1.5%)
Physical inactivity	1,342 (49.4%)	557 (48.8%)	785 (49.8%)
(Missing)	4	2	2

### 3.1 Prevalence of MetS

**Figure 2:**
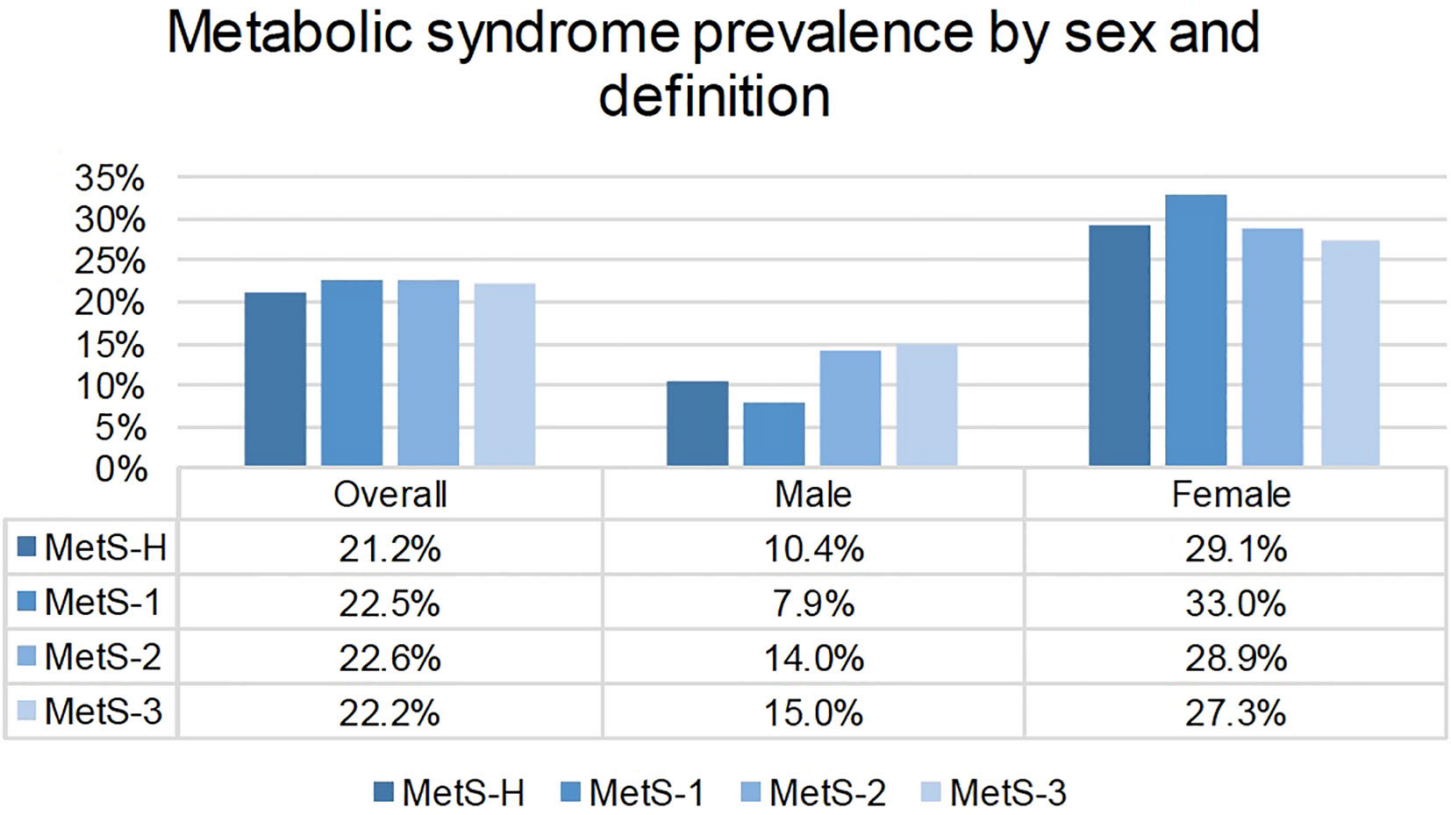
Prevalence of MetS by sex and definition.

The prevalence of MetS-H was 21.2%, which was greater in women than in men (29.1% vs 10.4%) (**Figure 2**. Prevalence of MetS by sex and definition). The age-standardized prevalence was 20.5% (95% CI 18.9-22.3). Among those with MetS-H, 79.4% were women, and 14.3% were younger than 40 years of age. The mean age of those with MetS-H was 53 years (SD 12). Among those who screened positive for MetS-H, 82.9% had elevated blood pressure, 90.7% had elevated waist circumference, and 81.7% had reduced HDL-C (**Table 3**).

**Table 3:** Proportion of participants meeting each clinical criteria, by MetS definition.

Clinical criteria	Overall N = 2,721	MetS-H N = 578	MetS-1 N = 611	MetS-2 N = 616	MetS-3 N = 603
Elevated Blood pressure	1,061 (39.0%)	479 (82.9%)	611 (100.0%)	588 (95.5%)	562 (93.2%)
SBP (mmHg) Median (IQR)	119 (107, 138)	140 (127, 155)	145 (134, 159)	146 (133, 162)	146 (133, 161)
DBP (mmHg) Median (IQR)	73 (63, 84)	85 (75, 94)	88 (81, 96)	88 (80, 97)	88 (79, 97)
On antihypertensive drug treatment	336 (12.4%)	184 (31.9%)	227 (37.2%)	272 (44.2%)	247 (41.0%)
Elevated Waist Circumference	1,223 (44.9%)	524 (90.7%)	611 (100.0%)	518 (84.1%)	492 (81.6%)
Waist circumference (cm) Median (IQR)	83.0 (74.0, 92.0)	96.0 (88.0, 102.0)	96.0 (89.0, 102.0)	95.0 (87.0, 102.0)	94.0 (86.0, 102.0)
Elevated fasting glucose*	293 (15.1%)	214 (50.0%)	142 (31.9%)	152 (33.4%)	145 (32.6%)
Fasting glucose (mg/dL) Median (IQR)	87 (82, 94)	98 (88, 111)	92 (86, 104)	93 (86, 105)	92 (86, 104)
Elevated nonfasting glucose**	26 (3.3%)	165 (21.0%)	17 (10.3%)	21 (13.1%)	18 (11.5%)
Non-fasting glucose (mg/dL) Median (IQR)	88 (82, 96)	93 (86, 102)	91 (85, 99)	92 (86, 105)	91 (85, 98)
Reduced HDL-C	1,106 (40.6%)	472 (81.7%)	334 (54.7%)	319 (51.8%)	306 (50.7%)
HDL-c (mg/dL) Median (IQR)	48 (41, 57)	43 (37, 48)	48 (41, 55)	48 (41, 56)	48 (41, 56)
Elevated Triglycerides	366 (13.5%)	258 (44.6%)	145 (23.7%)	148 (24.0%)	143 (23.7%)
Triglycerides (mg/dL) Median (IQR)	85 (61, 119)	137 (96, 189)	110 (84, 147)	111 (84, 147)	111 (84, 147)
Hospitalized for CVD	29 (1.1%)	16 (2.8%)	14 (2.3%)	16 (2.6%)	13 (2.2%)
Past Medical History	706 (25.9%)	334 (57.8%)	395 (64.6%)	517 (83.9%)	478 (79.3%)
Family History of CVD	1,185 (43.6%)	290 (50.2%)	312 (51.1%)	438 (71.1%)	434 (72.0%)
Smoking or Alcohol use	276 (10.1%)	36 (6.2%)	37 (6.1%)	37 (6.0%)	93 (15.4%)
High consumption of dietary fats/oils	2,363 (86.8%)	464 (80.3%)	486 (79.5%)	496 (80.5%)	552 (91.5%)

IQR, interquartile range, 25^th^ to 75^th^ percentile.

*Fasting blood glucose ≥100 mg/dL

**Nonfasting blood glucose ≥200 mg/dL

The prevalence of MetS-1 was 22.4% with an age-standardized prevalence of 21.7% (95% CI 20.0-23.5), 22.6% by MetS-2 definition, with an age-standardized prevalence of 21.8% (95% CI 20.2-23.7) and 22.1% by MetS-3 definition, with an age-standardized prevalence of 21.5% (95% CI 19.8-23.3) (**Figure 2**. Prevalence of MetS by sex and definition). Despite not using laboratory criteria, over 50% of participants who screened positive for MetS using the definitions of MetS-1, MetS-2, or MetS-3 had reduced HDL-C (range 50.7-54.7%) (**Table 3**).

By sex, the prevalence of metabolic syndrome was highest in males according to the MetS-3 definition (15%) versus the MetS-1 definition (7.9%) (**Figure 2**. Prevalence of MetS by sex and definition).

### 3.2 Pairwise comparison of MetS definitions

**Figure 3:**
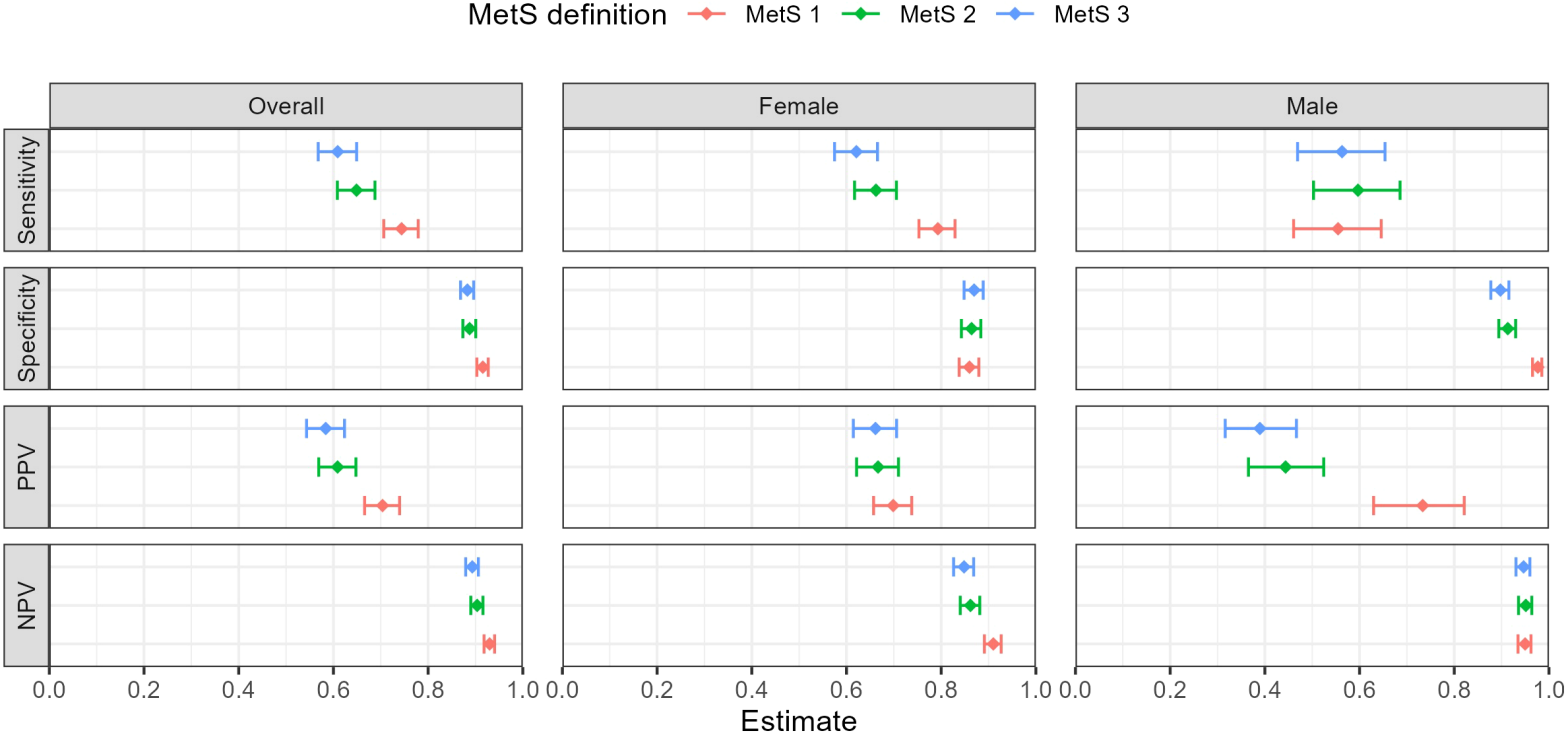
Diagnostic measures comparing MetS models to reference overall and by sex.

**Table 4** displays the 2x2 tables of MetS-H with each of the three new MetS definitions, and the Cohen’s kappa statistic for each pairwise comparison. Compared with MetS-H, MetS-1 had the highest sensitivity (74.4%, 95% CI 70.6-77.9%), followed by MetS-2 (64.9%, 95% CI 60.8-68.8%) and MetS-3 (60.9%, 95% CI 56.8-64.9%). MetS-1 had the highest specificity overall (91.6%, 95% CI 90.3-92.7), with others ≤89%. In terms of sex, MetS-1 had the lowest sensitivity for males, whereas it had the highest sensitivity for females. Overall, all nonlaboratory definitions had high negative predictive values (≥89%) but lower positive predictive values (range 58.4-70.4%) (**Figure 3**. Diagnostic measures comparing MetS models to reference overall and by sex). Compared with MetS-H, MetS-1 had substantial agreement in terms of the qualitative interpretation of the kappa statistics (64.6, 95% CI 61.0-68.2), whereas MetS-2 (52.4, 95% CI 48.3-56.4) and MetS-3 had moderate agreement (48.4%, 95% CI 44.2-52.6) (**Table 4**).

**Table 4:** Pairwise comparisons of MetS-H with nonlaboratory MetS definitions.

	MetS-H		
	Positive	Negative	Total
MetS-1			
Positive	430	181	611
Negative	148	1962	2110
Total	578	2143	2721
Cohen’s Kappa (95% CI)	–	–	64.6 (61.0, 68.2)
MetS-2			
Positive	375	241	616
Negative	203	1902	2105
Total	578	2143	2721
Cohen’s Kappa (95% CI)	–	–	52.4 (48.3, 56.4)
MetS-3			
Positive	352	251	603
Negative	226	1892	2118
Total	578	2143	2721
Cohen’s Kappa (95% CI)	–	–	48.4 (44.2, 52.6

### 3.3 Factors associated with metabolic syndrome

In multivariable regression including age, sex, education, poverty status and smoking status, only older age and female sex were associated with MetS-H. Compared with participants <30 years, there was a stepwise increase in MetS-H across age categories. Those aged 30–39 years were 2.55 (95% CI 1.64, 4.07) times more likely to have MetS-H, and those aged ≥60 years were 8.62 (95% CI 5.73-13.4) times more likely. Females were 2.47 (95%CI: 2.06, 2.98) times more likely to have MetS-H compared to men even after adjusting for all other factors (**Table 5**).

**Table 5:** Unadjusted and adjusted prevalence ratios of MetS-H from log-binomial regressions.

Characteristic	Unadjusted			Adjusted		
	PR	95% CI	p value	PR	95% CI	p value
Age group						
<30 years	Ref			Ref		
30–39 years	2.83	1.82, 4.44	<0.001	2.57	1.68, 4.04	<0.001
40–49 years	7.80	5.41, 11.69	<0.001	6.79	4.69, 10.20	<0.001
50–59 years	10.73	7.52, 15.95	<0.001	9.41	6.52, 14.10	<0.001
>60 years	9.63	6.74, 14.35	<0.001	8.59	5.89, 12.99	<0.001
Sex						
Male	Ref			Ref		
Female	2.79	2.33, 3.38	<0.001	2.47	2.07, 2.98	<0.001
Education						
≥secondary	Ref			Ref		
< secondary	2.61	2.25, 3.02	<0.001	1.07	0.92, 1.26	0.353
Poverty						
≤ $1 USD/day	Ref			Ref		
> $1 USD/day	1.09	0.94, 1.27	0.254	1.01	0.87, 1.15	0.927
Smoking						
Current/Former	Ref			Ref		
Never	1.5	1.09, 2.15	0.032	1.22	0.91, 1.72	0.210

PR, prevalence ratio; CI, confidence interval; ref, reference.

## 4 Discussion

We found that nearly one in every five Haitian adults (21.2%) living in Port-au-Prince had MetS, with a prevalence nearly 3 times greater in females than in males. This is especially notable given the overall young age of this cohort (mean age 42 years). Additionally, our data revealed that simplified definitions of MetS that do not require laboratory testing for glucose or lipids provide high specificity and sensitivity compared with gold-standard laboratory-based definitions and may be a simpler alternative for first level screening of MetS in low-income countries with similar populations. This is evidenced by the MetS-1 definition, which was found to have the highest sensitivity and specificity compared to the MetS-2 and MetS-3 definitions. While MetS-3 incorporates known risk factors of MetS, it was not shown to have a strong predictive value in comparison to the other definitions. This is potentially due to the fact that self-reported data may not be as objective as clinical data (such as blood pressure and waist circumference), and is subject to recollection bias (25).

The prevalence of MetS in Haiti falls within the range reported for other LICs. Studies in Nigeria, Sierra Leone, Guadeloupe, Martinique, and Botswana report low prevalence rates (range 8.5-28%) (26–32), whereas other settings, such as Central India and Puerto Rico, find alarmingly high estimates of over 40% (33, 34). The variation in prevalence could be attributed to the different criteria used, as there is no official standard definition. Compared with that in the United States, the prevalence of MetS in Haiti is much lower. Using NHANES data from 1988-2012, where the mean age of the cohort was 46.1 years, the prevalence in the United States was estimated to be 34.2% (35). The most significant components of metabolic syndrome in this cohort were elevated waist circumference (51.92%), elevated blood pressure (42.7%) and reduced HDL-C (44.0%), which are lower than what we found in Haiti.

However, our findings that elevated waist circumference and elevated blood pressure are the most common components of MetS in our cohort are consistent with trends in other LMICs. Studies in Martinique, Guadeloupe and Puerto Rico reported similar trends (32, 34).

Our study revealed that women had 3-fold higher rates of MetS than men did. This sex difference has been observed in other LMICs, with a MetS prevalence among females of 53% in Tanzania, 17.3% in Vietnam, 27% in Zambia and Zimbabwe, and 21.1% in Guadeloupe (28, 30, 31, 36). The reasons for sex differences may include that women in Haiti are more obese, as measured by both body mass index (BMI) and waist circumference (4). Elevated waist circumference, which contributes to insulin resistance (37, 38), was the predominant characteristic of those with MetS in our cohort (90.7% overall). This difference was greater in females than in males, which could be explained by differences in cultural practices and lower involvement in manual labor. In addition to female sex, our regression analysis revealed that older age was associated with MetS, similar to the findings of other studies in LMICs (27, 28).

Among the simplified definitions, MetS-1 was found to have the highest specificity and sensitivity compared with MetS-H. All the nonlaboratory definitions had high negative predictive values, suggesting that they may be useful for screening patients to rule out MetS. Notably, the high specificity and sensitivity of MetS-1 in Haiti are likely context dependent and directly related to the fact that elevated blood pressure and elevated waist circumference are the most common components of MetS. MetS-1 may not perform as well in other countries where diabetes or hyperlipidemia are the most common components. Other studies have developed nonlaboratory simplified definitions for MetS, including additional metrics such as heart rate variability and BMI, which are closely related to waist circumference (39–41). One study found that while using elevated BP and elevated height to waist ratio to estimate MetS prevalence had a high specificity (94.4%), it had a lower sensitivity (54.7%) (41). Another study identified BMI, very lower frequency of heart-rate variability, and BP were good predictors of MetS risk (40).

The accessibility of laboratory equipment remains an ongoing challenge in settings of extreme poverty and poor health system infrastructure, including urban Port-au-Prince. An estimated 47% of the global population lacks access to diagnostics, and the laboratory capacity for lipid monitoring is limited (42, 43) A recent study analyzed the availability of laboratory diagnostics in 10 LMICs, including Haiti, and reported limited access to essential diagnostic services, especially facilities for services (44). In low-resource settings without access to laboratory testing, measuring blood pressure and waist circumference alone may be a pragmatic alternative to screen individuals for MetS to identify those at a higher risk for cardiovascular events and death. Furthermore, both measurements can be performed in community settings by a vast cadre of providers spanning nurses and community health workers, which increases both access and coverage for screening. Individuals who screen positive for MetS can then undergo additional diagnostic testing. Longitudinal data are needed to assess if MetS is predictive of future CVD in Haiti.

Individuals screened with MetS-1 can receive targeted lifestyle counseling, such as dietary habits and physical activity, to help decrease their risk of developing these conditions later in life. As this is a screening tool, further evaluation should be done to confirm the diagnosis of MetS.

The strengths of this study include the use of a population-based cohort and research-grade blood pressure measurements. Limitations of the study include a lack of hemoglobin A1c data for diabetes screening and a lack of generalizability to nonurban settings. Another key limitation is that it does not evaluate the longitudinal outcomes of those that screened positive for MetS. Moreover, data are needed to determine whether MetS risk factors in Haiti are associated with incident heart disease, as predicted by studies in higher income settings. Potential future directions include validating MetS-1 in the community, and assessing the relationship between screening positive for MetS and long-term cardiovascular outcomes.

In conclusion, the prevalence of MetS in Port-au-Prince, Haiti, is 21.2% and is nearly three times greater in women. Consistent with other LMICs, the most prevalent components of MetS in the cohort were elevated blood pressure and elevated waist circumference. We were able to create a simplified definition of MetS that does not require laboratory values and has a high sensitivity and specificity that could be considered as a first-level screening tool in settings without laboratory capacity.

## Data Availability

The original contributions presented in the study are included in the article/**Supplementary Material**. Further inquiries can be directed to the corresponding author.
