# Supplementary material for: Capturing metabolic syndrome in low-resource settings: a case study in urban Haiti
Source: Front Endocrinol (Lausanne). 2025 Sep 11;16:1651058. doi: 10.3389/fendo.2025.1651058 (PMC12462050; doi:10.3389/fendo.2025.1651058)
Supplement: Supplementary file 1 [file Table1.docx]

**Supplementary Table 1: Comparison of nonlaboratory MetS definitions with MetS-H using sensitivity, specificity, positive predictive value and negative predictive value**

|  | | **Sensitivity** | | | **Specificity** | | | **PPV** | | | **NPV** | | |
| --- | --- | --- | --- | --- | --- | --- | --- | --- | --- | --- | --- | --- | --- |
| **Definition** | **Category** | **Estimate** | **95% CI** | | **Estimate** | **95% CI** | | **Estimate** | **95% CI** | | **Estimate** | **95% CI** | |
| MetS-1 | Overall | 0.74 | 0.71 | 0.78 | 0.92 | 0.90 | 0.93 | 0.70 | 0.67 | 0.74 | 0.93 | 0.92 | 0.94 |
| MetS-2 | Overall | 0.65 | 0.61 | 0.69 | 0.89 | 0.87 | 0.90 | 0.61 | 0.57 | 0.65 | 0.90 | 0.89 | 0.92 |
| MetS-3 | Overall | 0.61 | 0.57 | 0.65 | 0.88 | 0.87 | 0.90 | 0.58 | 0.54 | 0.62 | 0.89 | 0.88 | 0.91 |
| MetS-1 | Male | 0.55 | 0.46 | 0.65 | 0.98 | 0.97 | 0.98 | 0.73 | 0.63 | 0.82 | 0.95 | 0.93 | 0.96 |
| MetS-2 | Male | 0.60 | 0.50 | 0.69 | 0.91 | 0.89 | 0.93 | 0.44 | 0.37 | 0.52 | 0.95 | 0.94 | 0.96 |
| MetS-3 | Male | 0.56 | 0.47 | 0.65 | 0.90 | 0.88 | 0.92 | 0.39 | 0.32 | 0.47 | 0.95 | 0.93 | 0.96 |
| MetS-1 | Female | 0.79 | 0.75 | 0.83 | 0.86 | 0.84 | 0.88 | 0.70 | 0.66 | 0.74 | 0.91 | 0.89 | 0.93 |
| MetS-2 | Female | 0.66 | 0.62 | 0.71 | 0.86 | 0.84 | 0.88 | 0.67 | 0.62 | 0.71 | 0.86 | 0.84 | 0.88 |
| MetS-3 | Female | 0.62 | 0.57 | 0.67 | 0.87 | 0.85 | 0.89 | 0.66 | 0.61 | 0.71 | 0.85 | 0.83 | 0.87 |
| MetS-1 | Age <30 | 0.34 | 0.18 | 0.54 | 0.99 | 0.99 | 1.00 | 0.71 | 0.42 | 0.92 | 0.98 | 0.96 | 0.99 |
| MetS-2 | Age <30 | 0.28 | 0.13 | 0.47 | 0.99 | 0.98 | 1.00 | 0.53 | 0.27 | 0.79 | 0.97 | 0.96 | 0.98 |
| MetS-3 | Age <30 | 0.34 | 0.18 | 0.54 | 0.98 | 0.96 | 0.99 | 0.37 | 0.19 | 0.58 | 0.97 | 0.96 | 0.98 |
| MetS-1 | Age 30-39 | 0.76 | 0.62 | 0.87 | 0.95 | 0.93 | 0.97 | 0.65 | 0.52 | 0.77 | 0.97 | 0.95 | 0.98 |
| MetS-2 | Age 30-39 | 0.52 | 0.38 | 0.66 | 0.94 | 0.91 | 0.96 | 0.50 | 0.36 | 0.64 | 0.94 | 0.92 | 0.96 |
| MetS-3 | Age 30-39 | 0.50 | 0.36 | 0.64 | 0.92 | 0.89 | 0.94 | 0.41 | 0.29 | 0.54 | 0.94 | 0.91 | 0.96 |
| MetS-1 | Age 40-49 | 0.70 | 0.61 | 0.77 | 0.88 | 0.84 | 0.91 | 0.70 | 0.62 | 0.78 | 0.88 | 0.84 | 0.91 |
| MetS-2 | Age 40-49 | 0.61 | 0.52 | 0.69 | 0.85 | 0.81 | 0.89 | 0.63 | 0.54 | 0.71 | 0.84 | 0.80 | 0.88 |
| MetS-3 | Age 40-49 | 0.56 | 0.47 | 0.64 | 0.85 | 0.81 | 0.89 | 0.61 | 0.52 | 0.69 | 0.83 | 0.78 | 0.86 |
| MetS-1 | Age 50-59 | 0.80 | 0.74 | 0.86 | 0.84 | 0.79 | 0.88 | 0.77 | 0.70 | 0.83 | 0.87 | 0.82 | 0.90 |
| MetS-2 | Age 50-59 | 0.75 | 0.68 | 0.81 | 0.82 | 0.77 | 0.86 | 0.73 | 0.66 | 0.79 | 0.83 | 0.78 | 0.87 |
| MetS-3 | Age 50-59 | 0.72 | 0.64 | 0.78 | 0.83 | 0.78 | 0.87 | 0.74 | 0.66 | 0.80 | 0.82 | 0.77 | 0.86 |
| MetS-1 | Age 60+ | 0.78 | 0.71 | 0.84 | 0.78 | 0.73 | 0.82 | 0.66 | 0.59 | 0.72 | 0.86 | 0.82 | 0.90 |
| MetS-2 | Age 60+ | 0.68 | 0.60 | 0.75 | 0.66 | 0.61 | 0.71 | 0.53 | 0.46 | 0.59 | 0.79 | 0.73 | 0.83 |
| MetS-3 | Age 60+ | 0.61 | 0.54 | 0.69 | 0.69 | 0.63 | 0.74 | 0.52 | 0.45 | 0.59 | 0.76 | 0.71 | 0.81 |

**Supplementary Table 2: Multivariable regression for outcome of MetS-H using log-binomial, Poisson, and logistic models**

|  | **Log-Binomial** | | | **Poisson (robust variance)** | | | **Logistic** | | |
| --- | --- | --- | --- | --- | --- | --- | --- | --- | --- |
| **Characteristic** | **aPR** | **95% CI** | **p value** | **aPR** | **95% CI** | **p value** | **aOR** | **95% CI** | **p value** |
| Age group |  |  |  |  |  |  |  |  |  |
| <30 years | Ref |  |  | Ref |  |  | Ref | — | — |
| 30-39 years | 2.57 | 1.68, 4.04 | <0.001 | 2.55 | 1.64, 4.07 | <0.001 | 2.69 | 1.69, 4.37 | <0.001 |
| 40-49 years | 6.79 | 4.69, 10.20 | <0.001 | 6.87 | 4.63, 10.52 | <0.001 | 9.51 | 6.23, 15.0 | <0.001 |
| 50-59 years | 9.41 | 6.52, 14.10 | <0.001 | 9.27 | 6.25, 14.23 | <0.001 | 15.6 | 10.1, 24.8 | <0.001 |
| >60 years | 8.59 | 5.89, 12.99 | <0.001 | 8.62 | 5.73, 13.40 | <0.001 | 13.7 | 8.69, 22.1 | <0.001 |
| Sex |  |  |  |  |  |  |  |  |  |
| Male | Ref |  |  | Ref |  |  | Ref |  |  |
| Female | 2.47 | 2.07, 2.98 | <0.001 | 2.47 | 2.02, 3.05 | <0.001 | 3.5 | 2.77, 4.45 | <0.001 |
| Education |  |  |  |  |  |  |  |  |  |
| ≥secondary | Ref |  |  | Ref |  |  | Ref |  |  |
| < secondary | 1.07 | 0.92, 1.26 | 0.35 | 1.08 | 0.89, 1.31 | 0.35 | 1.13 | 0.89, 1.44 | 0.3 |
| Poverty |  |  |  |  |  |  |  |  |  |
| ≤ $1 USD/day | Ref |  |  | Ref |  |  | Ref |  |  |
| > $1 USD/day | 1.01 | 0.87, 1.15 | >0.9 | 1.02 | 0.85, 1.22 | >0.9 | 1.03 | 0.83, 1.29 | 0.8 |
| Smoking |  |  |  |  |  |  |  |  |  |
| Current/Former | Ref |  |  | Ref |  |  | Ref |  |  |
| Never | 1.22 | 0.91, 1.72 | 0.21 | 1.24 | 0.87, 1.84 | 0.21 | 1.37 | 0.90, 2.14 | 0.2 |

**Supplementary Table 3: Participants included with available data in the analyses compared to those with missing data or pregnancy.**

| **Characteristic** | **Overall Participants in Haiti CVD Cohort** | **Excluded in Analysis** | **Included in Analysis** | **p-value***^2^* |
| --- | --- | --- | --- | --- |
|  | N = 3,005*^1^* | N = 284*^1^* | N = 2,721*^1^* |  |
| MetS-H | 598 (21%) | 20 (24%) | 578 (21%) | 0.6 |
| Unknown | 200 | 200 | 0 |  |
| Sex |  |  |  | 0.8 |
| Male | 1,260 (42%) | 117 (41%) | 1,143 (42%) |  |
| Female | 1,745 (58%) | 167 (59%) | 1,578 (58%) |  |
| Age in years (range) | 40 (27, 55) | 36 (26, 49) | 41 (28, 55) | <0.001 |
| Age group |  |  |  | <0.001 |
| <30 yrs | 890 (30%) | 108 (38%) | 782 (29%) |  |
| 30-39 yrs | 569 (19%) | 54 (19%) | 515 (19%) |  |
| 40-49 yrs | 533 (18%) | 56 (20%) | 477 (18%) |  |
| 50-59 yrs | 499 (17%) | 39 (14%) | 460 (17%) |  |
| 60+ yrs | 514 (17%) | 27 (9.5%) | 487 (18%) |  |
| Education category |  |  |  | 0.4 |
| Greater or equal to secondary | 1,923 (64%) | 183 (67%) | 1,740 (64%) |  |
| Less than secondary | 1,073 (36%) | 92 (33%) | 981 (36%) |  |
| Unknown | 9 | 9 | 0 |  |
| Income |  |  |  | 0.079 |
| $1 - 10 USD/day | 360 (12%) | 22 (8.0%) | 338 (12%) |  |
| </= $1 USD/day | 2,105 (70%) | 198 (72%) | 1,907 (70%) |  |
| > $10 USD/day | 531 (18%) | 55 (20%) | 476 (17%) |  |
| Unknown | 9 | 9 | 0 |  |
| Fruit/Vegetable servings |  |  |  | 0.031 |
| <5 average servings/day | 2,974 (99%) | 270 (98%) | 2,704 (99%) |  |
| 5+ average servings/day | 20 (0.7%) | 5 (1.8%) | 15 (0.6%) |  |
| Unknown | 11 | 9 | 2 |  |
| Smoking |  |  |  | 0.3 |
| Current/Former | 221 (7.4%) | 15 (5.7%) | 206 (7.6%) |  |
| Never | 2,763 (93%) | 248 (94%) | 2,515 (92%) |  |
| Unknown | 21 | 21 | 0 |  |
| High Alcohol | 112 (3.7%) | 11 (4.1%) | 101 (3.7%) | 0.7 |
| Unknown | 17 | 17 | 0 |  |
| Physical Inactivity | 1,474 (49%) | 132 (48%) | 1,342 (49%) | 0.7 |
| Unknown | 14 | 10 | 4 |  |
